# Isolated granulocytic sarcoma of the pancreas: A tricky diagnostic for primary pancreatic extramedullary acute myeloid leukemia

**DOI:** 10.1186/1477-7819-10-13

**Published:** 2012-01-16

**Authors:** Mathieu Messager, David Amielh, Caroline Chevallier, Christophe Mariette

**Affiliations:** 1 Department of Digestive and Oncological Surgery, Centre Régional et Universitaire de Lille, Place de Verdun, 59037 Lille cedex, France; 2 Université Lille Nord de France, Place de Verdun, 59045, Lille cedex, France; 3 Inserm, UMR837, Team 5 « Mucins, epithelial differentiation and carcinogenesis » JPARC, Rue Polonovski, 59045 Lille cedex, France

**Keywords:** Granulocytic sarcoma, Chloroma, Myeloid tumor, Pancreas.

## Abstract

We report two clinical cases of primary granulocytic sarcoma of the pancreas that were diagnosed on the surgical specimen. Atypical clinical and morphological presentations may have lead to pretherapeutic biopsies of the pancreatic mass in order to indicate primary chemotherapy. Literature review of this rare clinical presentation may help physicians to anticipate diagnostic and therapeutic strategies.

## Background

Granulocytic sarcoma (GS) is an extramedullary solid tumor mass composed of immature myeloid cells [1]. GS is a rare manifestation of acute myeloid leukemia (AML) usually arising during or after the course of the disease, in up to 8% of patients in autopsy studies [2]. Occasionally, it can be the first and the only manifestation of AML, leading to diagnostic challenges. We report two exceptional cases of isolated pancreatic GS to focus physicians' attention to specific diagnostic and therapeutic strategies for a solid pancreatic mass.

## Cases presentation

**Figure 1:**
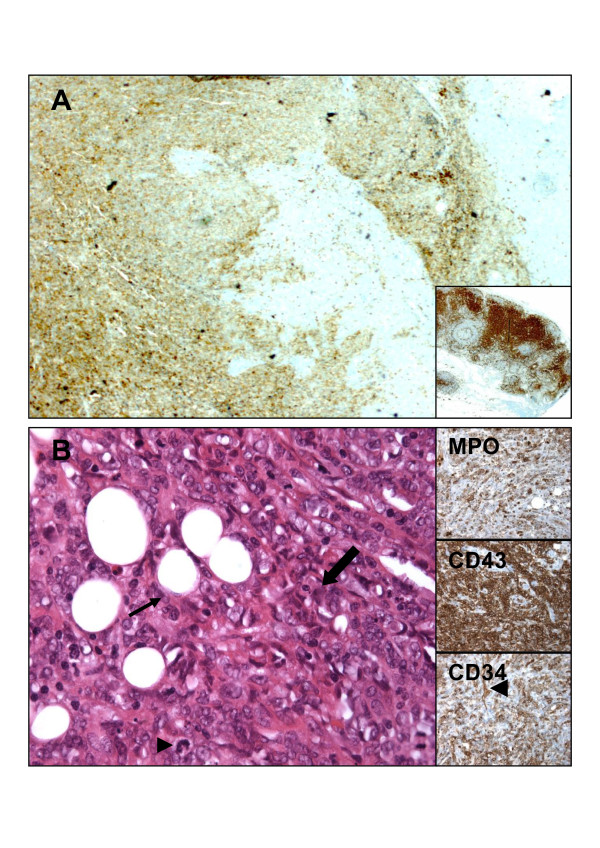
**Fixed, paraffin-embedded tissue sections of i) pancreatic invasion (A, case n°1, magnification at ×100) of medium sized cells, with CD43 positive expression signing myeloid lineage, inset shows contiguous lymph node with high CD43 expression (internal positive control of myeloid lineage); and of ii) omentum invasion (B, case n°2, hematoxylin and eosin staining, magnification at ×400) by myeloid-like cells, some with mitotic activity (arrow head), surrounding fat cells (arrow), inserts show myeloperoxydase (MPO), CD43, and CD34 expression (arrow head showing internal positive control with vessel)**.

The first patient was a 45-year-old woman, without significant comorbidity, who was referred to our institution for surgery. Epigastric pain with jaundice began one month previously without performance status alteration. Standard blood exams exhibited cholestasis (alkaline phosphatases 3.8 N, gama-glutamyl transpeptidases 37 N) and hyperamylasemia (1.9 N) with normal values of hemoglobin, white blood cells, platelets, carbohydrate antigen 19-9 (CA19-9) and carcinoembryonic antigen (CEA). Abdominal computed tomodensitometry (CT scan), magnetic resonance imaging (MRI) and endoscopic ultrasonography (EUS) of the pancreas all identified the distension of both the common bile duct (15 mm) and the Wirsung duct (6 mm), above a 28 × 20 mm irregular, hypoechoic and hypodense mass of the pancreatic head, without any lymph node or vascular invasion or distant secondary lesion detected. Based on the symptoms, a suspected diagnosis of pancreatic adenocarcinoma and a resectable mass, it was determined to proceed with primary surgery without obtaining preoperative sample biopsies. Curative whipple pancreaticoduodenectomy with regional lymphadenectomy was performed with no specific peroperative discovery and uneventful postoperative course. Histological examination of the surgical specimen revealed a pancreatic GS based on the presence of cells of myeloid lineage with positive immunostaining for CD43 myeloid-associated antigen (Figure 1A), whereas immunostainings for other myeloid markers (CD31, CD34, CD38, CD45, CD99, CD117), B-cell markers (CD20, CD79a), T-cell markers (CD3, CD4), commune B- and T-cell markers (CD30) and myeloperoxidase (MPO) were negative. Six weeks later, diffuse relapse occurred with the appearance of left cervical and multiple thoracic lymph nodes. After cervical biopsy, histological analysis confirmed recurrence with the same immunostaining profile. Brain tomodensitometry and bone marrow biopsy were normal. Cisplatin - cytarabin - dexamethasone-based chemotherapy was administered quickly, but the patient died due to disease dissemination one month later.

**Figure 2:**
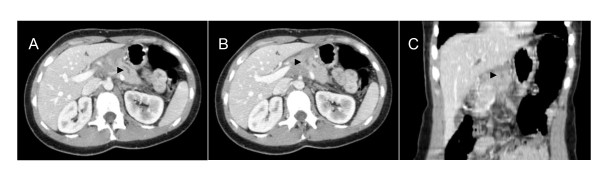
**Abdominal computed tomodensitometry, with injection of contrast product, portal sequence**. Axial (A) projection showing Wirsung dilatation (arrow head). Axial (B) and frontal (C) projections showing low density pancreatic mass (arrow heads), case n°2.

The second patient was a 19-year-old woman, without significant comorbidity or any alcohol consumption, who presented at our institution for epigastric pains combined with hyperamylasemia (1.7 N) and hyperlipasemia (7.8 N). Hemogram, hepatic enzymes, C-reactive protein, CEA and Ca 19.9 values were normal. The abdominal CT scan showed a 9-mm Wirsung duct dilation (Figure 2A) within the 30-mm mass of the pancreatic head (Figure 2B, C), the tumoral or inflammatory nature of which was uncertain. After conventional medical treatment for pancreatitis, the symptoms disappeared, allowing hospital discharge with additional morphological outpatient exams scheduled. Due to early recurrent epigastric pain episodes, combined with hyperlipasemia, she was re-admitted. EUS revealed an 11-mm celiac lymph node with a 9-mm Wirsung duct dilation and no clear pancreatic mass, whereas pancreatic MRI identified a moderately low signal intensity on T1-weighted images, middle-high signal intensity on T2-weighted images, and minimal enhancement on post-gadolinium images, consistent with the diagnosis of hypovascular solid tissues. Normal pentetreotide scintigraphy and the chromogranin A value ruled out the diagnosis of neuroendocrine tumor. Due to the absence of a clear diagnosis, persistent symptoms and the discordance between the exams that had been performed, the decision was made to proceed with a surgical exploration, revealing diffuse peritoneal carcinomatosis combined with an unresectable and inflammatory 30-mm pancreatic mass. Histological analysis of the pancreatic mass and peritoneal biopsies revealed extramedullar myeloid tumoral cells with immunohistochemistry positive for MPO, CD43, and CD34 (Figure 1B), as well as CD117 and CD45, and negative for CD79a, CD3, CD2, CD4, CD8 and CD68, leading to the diagnosis of pancreatic GS. The brain CT scan and bone marrow biopsy were normal. An induction cytarabin-based chemotherapy was begun quickly, leading to a complete morphological response after three consolidation cycles. Eight months later, left inguinal lymph node recurrence was diagnosed. Second-line amsacrine-cytarabin-based chemotherapy achieved a partial morphological response. Due to tumoral progression four months later, third-line clofarabine-based chemotherapy was administered with an optimal response that allowed bone marrow transplantation two months later. Diffuse peritoneal and hepatic recurrence was diagnosed based on PET scanning six months later, leading to palliation.

## Discussion

GS, also called chloroma, refers to the infrequent green color observed as a result of myeloperoxydase action in neoplastic cells [3]. GS usually occurs simultaneously or follows the onset of AML in 3-10% of patients [1,4]. Rarely, GS is the first manifestation of AML. GS may also be the first sign of transformation into AML in patients with myeloproliferative disorders or myelodysplasic syndrome [3]. Other common sites of origin are soft tissues, lymph nodes, skin and bones [5], with abdominal origin being very rare. Even if GS incidence is increasing due to prolonged leukemic remission of AML, pancreatic GS cases have rarely been reported in the literature. To our knowledge, 10 cases have been published (Table 1) [4,6-13], only four of which, in addition to the two reported in the present paper, were isolated pancreatic GS without bone marrow involvement [6,7,12,13]. Comparing with other published cases (Table 1), this work is to our knowledge, the first to describe two isolated pancreatic GS treated in a single center, with different therapeutic strategies, including a surgical approach. We also provided a complete follow-up for each case, critically analyzed the therapeutic strategies and highlighted the wandering diagnosis. Regarding other digestive locations, GS of the small intestine, colon and liver have been described, those situations being extremely rare [14,15].

**Table 1:** Clinical characteristics, treatment and outcomes of literature reports of pancreatic granulocytic sarcomas

Author/Year of report	Sex Age	Concomitant AML	Treatment	Response/Status
King *et al*./1987	F/36	No	Radiotherapy + CT (Daunorubicin, Cytarabine, Thioguanine)	CR
Moreau *et al*./1996	M/32	No	Duodenopancreatectomy + CT (Idarubin, Cytarabine)	CR after 2 years follow-up
Marcos *et al*./1997	F/37	Yes	None	Died after initial MRI
Ravandi - Kashani *et al*./1999	M/31	Yes	CT (Idarubicin, Cytarabine, All-trans retinoic acid)	CR, (follow-up unknown)
	F/61	Yes	CT (Idarubicin, Cytarabine, Lisofylline)	Recurrence, died
Servin-Abad *et al*./2003	M/64	In remission	CT (Unknown regimen)	CR, died of stroke
Breccia *et al*./2003	F/42	Yes	CT (Cytosine, Arabinoside, Idarubicin)+ BM allogarft	CR at 49 months from graft
Schäfer *et al*./2008	F/75	Yes	CT (Etoposide, Cytarabine, reduced dose Mitoxantrone)	Recurrence (7 months), died
Rong/2010	M/40	No	Duodenopancreatectomy + CT (Cytarabine based regimen)	CR, (follow-up unknown)
Li *et al*./2011	F/48	No	Distal pancreatectomy + splenectomy, patient refused adjuvant CT	Recurrence (2 months), died 3 months after surgery
Our study/2011	F/45	No	CT after duodenopancreatectomy (Cisplatin, Aracytine, Dexamethasone)	Early recurrence, died
	F/19	No	CT (Aracytine based regimen)	Recurrence (8 months), alive after BM transplantation (22 months follow-up)

AML: Acute Myeloid Leukemia; M: Man; F: Female; CT: Chemotherapy; CR: Complete Response; BM: Bone Marrow

GS can occur in patients of all ages with a focus on male patients (male:female ratio 1.2:1) during the last decades of life (median age is 56 years, range: 1 month - 89 years) [7,16]. Even if the overall prognosis of AML is favorable, the association with GS makes worsens the prognosis because only 24% of patients with GS will be alive 2 years after the initial diagnosis, with an overall median survival of 7 to 20 months [3,17].

Clinical behavior and response to therapy were not influenced by any of the following factors: age, sex, anatomic site, de novo presentation, histotype, phenotype or cytogenetic findings [18]. It remains uncertain what constitutes the best treatment in GS-associated AML patients [12]. However, high-dose chemotherapy and stem cell transplantation may benefit these patients, whereas radiation therapy or surgical resection have been found to be less effective [12].

These observations show that clinicians should think about pancreatic GS when the pancreatic mass develops during or after AML. However, in the cases reported here in which GS was the first and the only manifestation of AML, diagnosis is challenging. Because surgery is not required and may probably worsen the prognosis due to the delayed administration of induction chemotherapy, all efforts should be made to obtain pretherapeutic biopsies for a pancreatic mass, especially if all of the biological and morphological exam results are not typical and in agreement. The negative value of CA19.9 as well as the young age of our patients may have been warnings that indicate the value of EUS cytological examination for detecting differential diagnoses of pancreatic adenocarcinoma.

A positive diagnosis of GS is sometimes challenging and requires expert pathologists. Histological observation reveals myeloblats, promyelocytes and sometimes neutrophils. The definitive diagnosis of GS requires positive immunostaining for at least one of the myeloid-associated antigens (in decreasing frequency: CD68, MPO, CD43, CD45, CD117, CD99, CD33, CD34, CD13) associated with negative immunostaining for the lymphoid lineages (CD3 for T-cells and CD20 for B-cells) [1,12]. Major differential diagnoses are Hodgkin lymphoma, Burkitt lymphoma, large-cell lymphoma, and small round cell tumours. When a histological diagnosis of GS is made, bone marrow sampling is mandatory to assess the absence of AML.

The risk of metachronous AML occurrence in non-leukemic patients with GS is very high, with a median delay of 5 months; most patients will develop AML within 1 year [7,12]. Therefore, early intensive (induction/intensification) chemotherapy similar to that used to treat AML should be administered, even in GS patients who did not present AML upon initial diagnosis [3].

## Conclusions

The authors described two cases of isolated granulocytic sarcoma of the pancreas. The experience of these cases highlighted the difficulties of correct diagnosis and care. To conclude, pretherapeutic biopsies should be the cornerstone for the diagnosis of a pancreatic mass with atypical clinical presentation.

## Consent

Written informed consent was obtained from the patient for publication of this case report and the accompanying images. For the patient who died, consent was sought from the next of kin of the patient.

## Competing interests

The authors declare that they have no competing interests.

## Authors' contributions

Dr. DA and Dr. CC contributed to data collection. Dr. MM and Pr. CM contributed to writing the manuscript.
